# Comorbidities in lipedema: toward a systemic perspective – a narrative review

**DOI:** 10.1007/s10238-026-02157-9

**Published:** 2026-04-28

**Authors:** Elettra Fiengo, Andrea Sbarbati

**Affiliations:** 1 Private practitioner, Studio Iris, Rome, Italy; 2 Department of Neurological, Biomedical and Movement Sciences, Section of Anatomy and Histology, University of Verona, Verona, Italy

**Keywords:** Systemic disease, Connective tissue, Hypermobility, Mesenchymal stem cells, Chronic pain

## Abstract

Lipedema has historically been classified within obesity- and lymphedema-related frameworks, despite its distinct clinical features and frequent occurrence in individuals with normal or low body mass index. This narrative review examines whether the distribution of associated conditions reported in clinical cohorts is more consistent with a localized adipose-centered model or may suggest broader systemic involvement. Recent clinical and epidemiological studies have reported a clustering of manifestations affecting multiple biological domains in patients with lipedema, including connective tissue laxity and hypermobility, chronic venous disease, thyroid dysfunction and autoimmunity, endocrine–gynecological alterations, vitamin D deficiency, gastrointestinal disturbances, chronic widespread pain, and neuropsychological burden. Histopathological and microvascular investigations have also described alterations in extracellular matrix organization, stromal components, and endothelial structures within affected tissues. However, much of the available evidence derives from observational, cross-sectional, or survey-based studies, and potential confounding factors such as referral bias, obesity, and healthcare-seeking behavior should be considered when interpreting these associations. Taken together, these observations raise the possibility that lipedema may involve biological processes extending beyond adipose tissue alone. While current evidence does not yet establish a unified mechanism, the recurrent co-occurrence of connective, vascular, endocrine, and systemic features across multiple cohorts may be compatible with a broader vulnerability affecting connective tissue integrity and stromal microenvironment regulation. A multisystem perspective may therefore provide a useful conceptual framework for interpreting the clinical heterogeneity of lipedema and for guiding future mechanistic and longitudinal studies.

## Introduction

Lipedema was first described by Allen and Hines in 1940 as a condition characterized by a disproportionate accumulation of adipose tissue in the lower limbs, associated with pain and easy bruising. In their original description, the authors emphasized the marked female predominance and the lack of a significant response to caloric restriction, distinguishing the condition from simple obesity [1]. Historically, lipedema has also been frequently confused with lymphedema, despite the absence of primary lymphatic dysfunction in many affected individuals and the occurrence of the disease across a wide range of body mass indices.

In recent years, research on lipedema has expanded substantially, contributing to a more precise clinical and histopathological characterization of the disease [2]. The most widely accepted interpretative framework currently describes lipedema as a disorder primarily affecting subcutaneous adipose tissue, influenced by hormonal factors and frequently associated with microvascular and venous alterations [3, 4]. Beyond adipocyte abnormalities, several histopathological studies have demonstrated extracellular matrix remodeling and interstitial fibrosis within lipedematous tissue, with increased stromal components and alterations in connective tissue architecture [5]. More recently, Michelini et al. (2025) documented structural alterations of endothelial cells in the capillaries of lipedematous adipose tissue, highlighting direct involvement of the microvascular compartment and the stromal microenvironment [6], which may contribute to tissue stiffness, pain, and progressive functional impairment.

As early as 2010, Child et al. [7] described lipedema as an inherited condition, observing a pattern compatible with autosomal dominant transmission with sex limitation in several affected families. The frequent familial aggregation reported across multiple clinical cohorts suggests the presence of a genetic predisposition. Genome-wide association studies in the “UK Lipoedema” cohort identified multiple susceptibility signals across different loci, without evidence of a single causative gene, supporting a complex polygenic architecture [8]. In parallel, candidate-gene and computational studies have suggested possible involvement of steroid metabolism pathways, including AKR1C1, implicated in progesterone and estrogen metabolism [9]. Exploratory studies have also reported possible somatic mutations within lipedematous tissue [10], indicating that local genetic alterations may contribute to phenotypic variability. Collectively, these findings support a multifactorial genetic predisposition interacting with hormonal and environmental factors, rather than a monogenic disease model.

The adipose–vascular paradigm has represented a fundamental advance, allowing lipedema to be distinguished from obesity and primary lymphedema, facilitating shared diagnostic criteria and guiding both conservative and surgical management. Recent international consensus documents acknowledge that lipedema is frequently associated with obesity, endocrine alterations, connective tissue disorders, and a significant impact on mental health and quality of life [11]. These statements also highlight the limited clinical utility of staging systems based exclusively on morphological criteria. In this context, Al-Ghadban et al. (2025) proposed a revised staging approach integrating pain, body composition, and muscular involvement, underscoring the need to move beyond a purely volumetric interpretation [12].

In parallel, an increasing number of studies have documented a broad spectrum of associated manifestations involving multiple biological domains, including the vascular compartment, connective tissue, endocrine–reproductive axis, gastrointestinal function, and the neuropsychological sphere [13]. These features are typically described as comorbidities—coexisting conditions considered conceptually separate from a predominantly adipose pathological core. However, the recurrent clustering of manifestations across functionally distinct systems raises a critical interpretative question: do these associations represent coincidental co-occurrence, or do they reflect organ-specific expressions of a shared biological vulnerability?

While the adipose–vascular model has been essential for clinical recognition and management, the growing heterogeneity of associated manifestations and the frequent dissociation between morphological stage and symptom severity suggest the need for an expanded interpretative framework. In particular, stromal remodeling and fibrosis—already documented at the histopathological level—may represent key elements for understanding the complexity of the lipedema phenotype beyond adipose expansion alone. This narrative review examines the distribution of associated conditions reported in lipedema and discusses whether their recurrent multisystem patterns may suggest biological links that extend beyond a purely adipose-centered interpretation.

## Comorbidities in lipedema: A multisystemic distribution

A review of major published cohorts highlights the recurrent presence of manifestations affecting multiple biological domains, including connective tissue laxity and hypermobility, multisite joint pain and functional instability, chronic venous disease with stage-dependent progression, thyroid dysfunction and autoimmunity, ovarian axis alterations including polycystic ovary syndrome (PCOS), vitamin D deficiency, gastrointestinal disturbances, and mood and attentional symptoms. Despite methodological heterogeneity, these findings consistently document multisystemic involvement across independent cohorts. Importantly, however, the presence of these conditions within lipedema cohorts does not necessarily indicate an increased prevalence compared to the general population, as direct comparative epidemiological data are often lacking. The distribution of associated conditions across domains is summarized in Table 1.

### Hypermobility and connective tissue

Signs of connective tissue laxity have been reported in multiple clinical studies. In our observational study on lipedema and Hypermobility Spectrum Disorders (Fiengo & Sbarbati, 2025) [14], 44% of patients reported hypermobile body regions, and approximately 60% reported joint hypermobility during childhood. These findings are consistent with previous observations (Torre 2017) [15] and suggest that hypermobility traits may be underestimated in adulthood, possibly due to progressive fibrosis and adipose proliferation reducing Beighton score sensitivity.

Simarro Blasco et al. (*n* = 1803) [13] reported ligamentous laxity in 95.8% of evaluated patients. In the Italian cohort described by Patton et al. (*n* = 360) [16], joint hypermobility (Beighton ≥ 5) was present in 31.59% (73/232), with dislocations or sprains reported in 20.9% and fractures in 29%. Aday et al. (2024) [17] found significantly higher odds of joint hypermobility in women with lipedema compared with controls (OR 12.88; *p* < 0.0001).

Beyond the musculoskeletal system, Nemes et al. (2019) [18] identified alterations in mitral annular dynamics using three-dimensional speckle-tracking echocardiography, suggesting that connective tissue involvement may extend beyond the peripheral compartment. Taken together, these data indicate that tissue hypercompliance represents a recurrent feature in a substantial subset of patients.

While joint hypermobility and connective tissue laxity are consistently reported across lipedema cohorts, the relationship with defined connective tissue disorders such as hypermobile Ehlers–Danlos syndrome (hEDS) remains unclear, as it has not been systematically investigated and cannot be established based on the currently available evidence.

### Pain

Joint and periarticular pain is among the most frequently reported manifestations and does not consistently correlate with adipose volume. In our observational study, multisite pain involved ankles (70%), knees (56%), cervical spine (66%), sacral region (46%), and feet (55%) [14]. Simarro Blasco et al. [13] reported bilateral trochanteric pain in 97.4% of cases. The convergence of pain, instability, and connective tissue laxity across cohorts suggests that musculoskeletal symptoms cannot be interpreted solely as mechanical overload.

In a German cohort, approximately 22.5% of patients reported migraine, with improvement after liposuction in 68% of cases [5]. Erden et al. (2025) [19] reported a mean VAS score of 6.38 ± 1.71 and a neuropathic pain prevalence of 43.3%, with significantly higher pain intensity and reduced quality-of-life scores in affected individuals. Histological findings by Chakraborty et al. (2022) [20] demonstrated reduced nerve fiber density and features of neurogenic inflammation. However, QST studies in non-obese patients identified selective sensory alterations without generalized systemic sensitization [21], suggesting that extracellular matrix changes and increased tissue stiffness may contribute to pressure transmission and pain perception. The mechanisms underlying pain remain incompletely defined.

Pain in lipedema may extend beyond the lower limbs and involve multiple anatomical regions, including the upper extremities, joints, and spine, reflecting a heterogeneous distribution that does not necessarily follow a strictly region-specific pattern.

### Fibromyalgia

Fibromyalgia has been diagnosed in 35% of patients according to ACR 2016 criteria, with self-reported prevalence of 17% in national surveys [22]. Bolkan Günaydın et al. (2025) [23] described frequent features compatible with lipedema in patients with fibromyalgia, highlighting a relevant clinical overlap. Although causality remains unproven, the intersection between these conditions suggests possible shared biological pathways.

### Endocrine and thyroid alterations

Endocrine alterations constitute one of the most consistent associated clusters. In the cohort described by Patton et al. [16], hypothyroidism was present in 22.5% of patients, increasing to approximately 30% when including prior diagnoses, while autoimmune thyroiditis was reported in 35.5% and vitamin D deficiency in 84.6%. Bauer et al. [5] reported hypothyroidism in 35.9%, and Kruppa et al. [24] in 31%. Simarro Blasco et al. [13] described thyroid disease in 59.5% of patients, with thyroid nodules detected in 88.7% of those undergoing ultrasound. Despite methodological variability, thyroid-related conditions have been reported across independent cohorts, with heterogeneous presentations.

### Gynecological domain

Reproductive and gynecological alterations are also frequently reported. In the cohort described by Patton et al. [16], menstrual irregularities were present in 32.5% of patients, PCOS in 17.1%, and self-reported polycystic ovaries in 19.2%, with endometriosis (4.2%) and fertility problems (9%) also documented. Simarro et al. [13] reported inflammatory ovarian–uterine symptoms in 76% of cases. The scoping review by Viana et al. (2026) [25], including 25 studies across ten countries, highlighted recurrent associations with steroid-dependent gynecological conditions and proposed a broader endocrine–metabolic characterization of lipedema.

### Gastrointestinal system

Intestinal disorders were reported in over 40% of cases in the cohort described by Patton et al. [16], with gastric disturbances in 32.9%. In our observational study, 30% of pediatric patients reported abdominal pain and 50% of adults reported digestive difficulties [14]. Simarro Blasco et al. [13] reported a high frequency of clinical suspicion of increased intestinal permeability, although without standardized diagnostic confirmation. Overall, gastrointestinal symptoms appear recurrent.

### Neuropsychological disturbances

Mood disorders were reported in over 40% of patients in the Italian cohort [16], with increasing prevalence across stages. Swiss and Polish studies reported depressive symptoms in 59–64% of women with lipedema [26, 27], while Ghods and Kruppa [24] documented clinical depression in 25.5%. Amato et al. [28] reported positive ADHD screening (ASRS) in 77% of patients compared with 54% of controls (RR 1.424; *p* < 0.0001). In a cohort of 511 patients, Hamatschek et al. (2022) [29] reported high psychological burden and significant work limitations, alongside a high prevalence of hypothyroidism and low prevalence of type 2 diabetes, suggesting a metabolic profile distinct from simple obesity.

Cognitive complaints, often described as “brain fog,” including difficulties in concentration and mental fatigue, have been reported in some patients, although their prevalence and underlying mechanisms remain poorly defined.

### Venous and lymphatic involvement

Chronic venous disease was present in 71.9% of patients in the Patton cohort [16] and reached 86.2% in a Swiss referral center. Simarro Blasco et al. [13] described venous insufficiency and tissue congestion with possible progression toward mixed lipo-lymphatic phenotypes. Aday et al. [17] reported markedly increased odds of edema in heat, easy bruising, gait alterations, varicose veins, and fatigue compared with controls.

Lymphoscintigraphy and functional imaging studies have demonstrated delayed lymphatic drainage in subsets of patients, particularly in advanced stages or mixed forms [30]. Non-contrast MR lymphography [31], interstitial fluid analyses [32], and 3T MR lymphangiography [33] have identified alterations in the fluid–stromal microenvironment without reproducing classical primary lymphedema patterns. Overall, lymphatic dysfunction appears to act as a modulatory factor in disease progression rather than as a primary defect. These alterations may contribute to commonly reported sensations of tissue heaviness and swelling-like symptoms, which do not necessarily correspond to classical edema but may reflect changes in interstitial fluid dynamics and tissue properties.

Immune-related mechanisms may also be involved and could contribute to clinical features such as allergies, asthma, and sensitivity to histamine or nickel, as reported in some clinical cohorts [13]. In this context, emerging hypotheses have suggested a potential role of mast cell involvement in lipedema, although current evidence remains limited and largely hypothesis-generating [34].

**Table 1:** Multisystemic distribution of conditions associated across major lipedema cohorts

Domain	Key manifestations reported	Representative cohorts	Observed pattern
Connective tissue	Hypermobility, ligamentous laxity, valvular changes	[13, 14, 16–18]	High recurrence across independent cohorts
Pain	Multisite pain, neuropathic features, migraine	[5, 13, 19–21]	Not proportional to adipose volume
Endocrine	Hypothyroidism, autoimmune thyroiditis, vitamin D deficiency	[5, 13, 16, 24]	Frequent endocrine clustering
Gynecological	PCOS, menstrual irregularities, endometriosis	[13, 16, 25]	Steroid-dependent association
Gastrointestinal	Abdominal pain, digestive disturbances	[13, 14, 16]	Recurrent but under-investigated
Neuropsychological	Depression, ADHD traits, QoL impairment	[16, 24, 26–29]	Stage-related burden
Venous/Lymphatic	CVD, fluid alterations, delayed drainage	[13, 16, 17, 30–33]	Modulatory rather than primary defect
Immune-related	Allergies, asthma, histamine/nickel sensitivity	[13, 34]	Reported in some cohorts; not systematically assessed

## Discussion

**Fig. 1:**
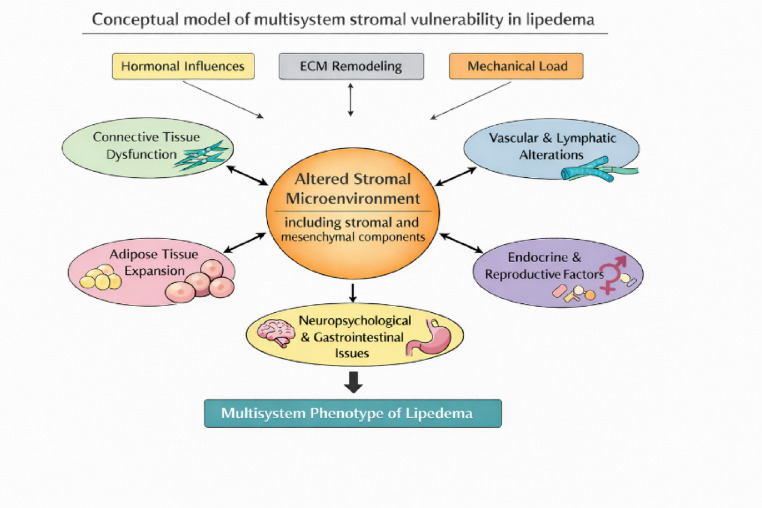
Proposed conceptual framework of lipedema as a multisystem disorder

A review of the major published cohorts indicates that conditions associated with lipedema involve multiple biological domains, including connective tissue, vascular and lymphatic compartments, the endocrine–reproductive axis, the gastrointestinal tract, and the neuropsychological sphere [13, 16, 26]. Although many of these manifestations have been individually reported in previous clinical studies, they are generally described as comorbidities coexisting with a disease considered primarily adipose in nature [4, 16]. However, the recurrence of similar clusters across independent cohorts suggests that this distribution may be unlikely to represent a purely coincidental aggregation. To facilitate interpretation of these observations, a conceptual framework integrating the potential relationships between stromal alterations and multisystem manifestations in lipedema is presented in Fig. 1.

The diagram summarizes a hypothesis-generating model in which alterations of the stromal microenvironment, including stromal and mesenchymal components, may contribute to adipose, connective, vascular–lymphatic, endocrine, gastrointestinal, and neuropsychological manifestations reported in lipedema.

The adipose–vascular model has provided an essential interpretative framework for the clinical definition of lipedema and has been instrumental in distinguishing it from obesity and primary lymphedema. International consensus statements continue to emphasize the role of adipose tissue expansion, microvascular fragility, and hormonal influences as central components of the disease [3, 4, 11]. This model has undoubtedly enabled standardized staging systems and guided both conservative and surgical interventions. Nevertheless, several features reported in the literature remain only partially explained within a strictly localized paradigm. These include the dissociation between morphological stage and symptom severity, the presence of severe pain in early stages with minimal vascular alterations, and the persistence of systemic manifestations after adipose reduction procedures [26, 29].

Importantly, available data do not support the classification of lipedema as a primary systemic inflammatory disease [5, 6]. Studies investigating inflammatory markers have not consistently demonstrated a generalized inflammatory state. Rather, inflammatory changes described at the tissue level may reflect localized responses or secondary phenomena within a broader stromal context. This interpretation aligns with histopathological evidence of extracellular matrix remodeling and fibrosis, which may alter tissue biomechanics and fluid dynamics without necessarily implying systemic inflammation. Alternative systemic hypotheses have been proposed, including models suggesting that circulating endotoxins, intestinal permeability, and complement activation may contribute to local adipose tissue expansion [35]. While such models may account for specific aspects of the disease, they may not fully account for the breadth of clinical variability observed in lipedema. In this context, different mechanisms may coexist and contribute in a context-dependent manner within a shared systemic vulnerability.

In parallel, experimental studies have reported alterations in adipose-derived stem cells in lipedema, including differences in adipogenic response compared to controls [36]. Although these findings are derived from in vitro analyses, they support the plausibility of altered stromal regulation within a broader systemic context.

Evidence from other fields has suggested that stromal and mesenchymal components may be involved in the pathophysiology of proliferative conditions, including endometriosis, through their role in tissue regeneration, immune modulation, and lesion formation. Although these findings are not specific to lipedema, they are consistent with the concept of a shared stromal vulnerability affecting multiple domains.

More broadly, several studies have highlighted the role of stromal and mesenchymal components in regulating tissue microenvironments and modulating cellular behavior across different biological systems [37, 38]. Although these findings are not specific to lipedema, they further support the plausibility of a shared stromal vulnerability affecting multiple domains.

The recurrent presence of features compatible with connective tissue vulnerability—including hyperlaxity, stromal expansion, and interstitial alterations—has been documented across multiple studies [13, 14, 17]. While previous authors have primarily interpreted these findings as associated conditions, their systematic recurrence across functionally distinct systems raises the possibility of a shared biological substrate. In this perspective, adipose tissue expansion would represent a predominant site of phenotypic expression rather than the exclusive pathological core.

Based on the available evidence, a conceptual model can be hypothesized in which lipedema represents a peripheral manifestation of a broader dysfunction of the stromal microenvironment, potentially involving extracellular matrix regulation, mechanotransduction, and interstitial fluid homeostasis. Hormonal and mechanical factors may act as modulators of clinical expression, contributing to the phenotypic variability observed among patients with similar morphological staging.

Within such a framework, joint and neuropathic pain, endocrine alterations, gastrointestinal disturbances, vascular and lymphatic involvement, and neuropsychological impact may be reconsidered not merely as independent comorbidities, but potentially as organ-specific expressions of a shared regulatory vulnerability. Expanding the current paradigm does not negate the validity of the adipose–vascular model; rather, it situates it within a broader interpretative structure. The existing model remains fundamental for clinical management, yet it may represent one descriptive layer of a more complex biological phenomenon. If confirmed through prospective and mechanistic studies, this systemic stromal perspective could refine disease classification and patient stratification, supporting integrated therapeutic approaches that address not only adipose tissue volume but also connective, endocrine, and neurofunctional dimensions. Further research employing standardized diagnostic criteria and multidimensional assessments will be essential to clarify the mechanisms underlying the observed multisystemic distribution.

## Limitations

This narrative review has several limitations. The available studies are heterogeneous in diagnostic criteria, study design, and cohort selection, which may influence the comparability of reported findings. Most cohorts derive from specialized referral centers and rely largely on observational data, and in some cases self-reported information, potentially introducing selection bias and confounding factors. In addition, the present analysis represents a narrative synthesis rather than a systematic review or meta-analysis and therefore cannot quantify the strength of associations between lipedema and the conditions described. Consequently, the systemic interpretative framework proposed here should be considered hypothesis-generating and requires confirmation through prospective and mechanistic studies.

## Conclusion

The currently available clinical and biological evidence suggests that manifestations associated with lipedema may not occur randomly, but instead tend to involve recurrently distinct functional domains. The repetition of this pattern across independent cohorts, investigated using different methodologies and within diverse geographical contexts, makes a strictly localized interpretation of the disease increasingly difficult to fully reconcile with the available observations. The adipose–vascular paradigm has represented a fundamental step in the clinical definition of lipedema and in its distinction from obesity and lymphedema. However, the frequent dissociation between morphological stage and symptom severity, the presence of neuropathic pain, histologically documented stromal alterations, endocrine and gynecological involvement, changes in the fluid–interstitial microenvironment, and the persistence of symptoms following adipose tissue volume reduction suggest that the adipose compartment may not represent the sole pathogenic driver of the condition. Within this context, lipedema may potentially represent a predominant phenotypic expression of a broader systemic vulnerability involving connective tissue and the stromal microenvironment. Alterations of the extracellular matrix, stromal remodeling, hormonal modulation, and alterations in stromal and mesenchymal components may act as interconnected elements of a shared biological substrate, in which adipose tissue represents a preferential site of manifestation rather than the only target. From this perspective, conditions commonly described as “comorbidities” may potentially reflect organ-specific manifestations within a broader biological terrain. Pain, endocrine alterations, connective tissue involvement, vascular–lymphatic changes, and neuropsychological manifestations may therefore represent differentiated phenotypic expressions of a systemic vulnerability affecting the stromal microenvironment.

Reductive surgery may decrease volumetric burden and improve certain symptomatic aspects; however, it does not exhaust the physiological complexity of the disorder nor necessarily address the biological substrate sustaining its multisystemic expression. Recognizing lipedema as a complex biological disorder with predominant adipose expression may therefore encourage a shift from morphology-based assessment toward integrated pathophysiological interpretation, from visual staging toward systemic phenotypic stratification, and from compartmentalized management toward genuinely multidisciplinary care. The adipose–vascular paradigm has enabled the clinical recognition of lipedema. A broader systemic perspective may enable a deeper understanding of the condition.

## Key References

- Cifarelli V. Lipedema: Progress, Challenges, and the Road Ahead. Obes Rev. 2025;26:e13953.This comprehensive recent review summarizes current knowledge on lipedema pathophysiology and highlights the need for broader conceptual frameworks that go beyond an exclusively adipose-centered interpretation of the disease.
- Michelini S, Greco S, Vaia N et al. Endothelial cell alterations in capillaries of adipose tissue from patients affected by lipedema. Obesity (Silver Spring). 2025;33:695–708.This histopathological study demonstrates structural alterations of endothelial cells within lipedematous adipose tissue, supporting the role of microvascular dysfunction and stromal microenvironment alterations in lipedema.
- Kruppa P, Crescenzi R, Faerber G et al. Lipedema World Alliance Delphi consensus-based position paper on the definition and management of lipedema. Nat Commun. 2026;17:427.This international consensus document provides an updated clinical framework for lipedema diagnosis and management based on expert agreement and represents a key reference for current diagnostic criteria.
- Simarro Blasco JL, Michelini S, Andrés-Gasco M et al. Clinical signs at diagnosis and comorbidities in a large cohort of patients with lipedema in Spain. Biomedicines. 2025;13:3049.This large cohort study provides important epidemiological data on the clinical presentation and associated conditions in lipedema, confirming the frequent coexistence of multiple systemic manifestations.
- Bauer AT, von Lukowicz D, Lossagk K et al. New insights on lipedema: the enigmatic disease of the peripheral fat. Plast Reconstr Surg. 2019;144:1475–1484.This review highlights the role of alterations in the adipose stromal microenvironment, including vascular dysfunction and progenitor cell dysregulation, suggesting that lipedema cannot be explained solely by adipocyte hypertrophy.
- Fiengo E, Sbarbati A. Lipedema and hypermobility spectrum disorders sharing pathophysiology: a cross-sectional observational study. J Clin Med. 2025;14:7195.This study explores the overlap between lipedema and hypermobility spectrum disorders and highlights shared connective tissue features supporting a broader systemic vulnerability framework.
- Dinnendahl R, Tschimmel D, Löw V et al. Non-obese lipedema patients show a distinctly altered quantitative sensory testing profile with high diagnostic potential. Pain Rep. 2024;9:e1155.This study demonstrates altered sensory profiles in non-obese patients with lipedema using quantitative sensory testing, highlighting the relevance of neuropathic and sensory components in the disease.
- Viana DPDC, Invitti AL, Schor E. Lipedema in women and its interrelationship with endometriosis and other gynecologic diseases: a scoping review. Biomedicines. 2026;14:122.This review explores the relationship between lipedema and gynecological disorders, particularly endometriosis, suggesting shared hormonal and stromal mechanisms.

## Data Availability

No datasets were generated or analysed during the current study.
